# A Review of Solar-Coupled Phase Change Materials in Buildings

**DOI:** 10.3390/ma16175979

**Published:** 2023-08-31

**Authors:** Shahid Aziz, Tariq Talha, Abdur Rehman Mazhar, Junaid Ali, Dong-Won Jung

**Affiliations:** 1 Department of Mechanical Engineering, Jeju National University, 102 Jejudaehak-ro, Jeju-si 63243, Republic of Korea; shahid@jejunu.ac.kr; 2 Institute of Basic Sciences, Jeju National University, 102 Jejudaehak-ro, Jeju-Si 63243, Republic of Korea; 3 College of Electrical & Mechanical Engineering, National University of Sciences & Technology, Islamabad 47301, Pakistan; 4 Optoelectronics Research Laboratory, Department of Physics, COMSATS University Islamabad, Islamabad 45500, Pakistan; 5 Faculty of Applied Energy System, Major of Mechanical Engineering, Jeju National University, 102 Jejudaehak-ro, Jeju-si 63243, Republic of Korea

**Keywords:** solar, phase change materials (PCMs), indoor heating, indoor cooling, thermal storage, active systems, passive systems

## Abstract

Buildings use a significant percentage of the total energy consumed worldwide. Striving for energy conservation within buildings is of prime concern for researchers. Hence, scientists are aggressively exploring new energy storage and supply methods to reduce exorbitantly fluctuating energy demands and increase the share of renewable energy in building energy consumption. Solar systems that incorporate phase change materials (PCMs) for thermal storage have significant potential to serve in this context. These systems are not yet able to endure the significant energy demands, but they are being continually improved. The aim of this paper is to explore the existing solar PCM systems that are being studied or that are installed for use in indoor heating/cooling. As per the outcome of this systematic review, it has been observed that when coupled with solar thermal energy, the configuration of PCMs can either use passive or active techniques. Passive techniques are usually less efficient and more costly to implement in a building structure, resulting in active heat exchangers being widely implemented with better technical and economic results. At the same time, it has been observed that for most domestic buildings, organic PCMs with phase change temperatures of up to 42 °C and thermal conductivities of up to 0.56 W/m.K are most suitable for integration in solar thermal energy production. Hybrid systems are also commonly used for larger commercial buildings, in which the solar PCM system (SPCMS) provides a fraction of the total load. Additionally, the Stefan number is the most common technical parameter that is used to assess this performance, along with the effective thermal conductivity of the PCM after using enhancement techniques. The key economic indicator is annual savings per year, with most SPCMSs having a payback period of between 6 to 30 years. This review provides designers and researchers with key insights in terms of formulating a basis in the domain of coupling PCMs with solar thermal energy, especially within non-industrial buildings.

## 1. Introduction

Worldwide energy consumption is increasing exponentially with the ever-increasing population and aggressive industrialization of recent years. We stand in an era where natural energy resources are depleting at an expeditious rate, and their demand is increasing exponentially. In industrial countries, one-third of total energy is consumed by HVAC systems only [1]. This leads researchers to devise methods for providing alternative energy resources for heating and cooling requirements. Numerous studies have been conducted on the use of renewable energies in an optimized manner to share the burden of energy consumption in buildings. Among all renewable energies, solar energy is the most researched because it is clean, renewable, easy to harness, and safe [2]. The energy from the sun can be directly employed to heat buildings or, alternatively, it can be concentrated on thermal collectors that, in turn, heat a carrying medium like water, carrying the heat through the system and supplying it to the desired location. The use of thermal collectors results in a higher absorption of solar energy when compared to direct heating and, hence, is preferred [3]. Solar thermal energy can also be harnessed for cooling systems as well. Selvaraj et al. [4] devised a vapor absorption cycle based on solar photovoltaic (PV) panels to compress a refrigerant thermally in the generator section of a vapor absorption cycle. Thus, a compressor, which is the component that carries the major load in a refrigeration cycle, was replaced by a renewable energy source to achieve clean and free cooling. The COP of the modified configuration was found to have increased by 0.04 in comparison to the commercial AC absorption refrigeration cycle, with a payback time of 10.2 years.

A significant amount of energy is consumed by buildings to provide thermal comfort for their inhabitants, and this contributes to serious climatic changes. Hence, there is an urgent need to reduce the thermal energy consumption of this residential sector. A significant research effort has been invested in achieving zero-energy buildings, and thermal energy storage (TES) seems to be the best way to achieve this goal by decoupling the demand and supply [5]. In today’s world, the usefulness of TES is well established, and it seems to provide an optimistic prospect towards achieving a low-carbon future. TES technology is described as an initiative towards a reduction in energy consumption in buildings. It aims to reduce the urban heating island (UHI) effects in cities and to increase energy efficiency and thermal comfort by balancing the energy demand between day and night [6]. Energy can be stored in either physical form or chemical form. Physically, energy is stored as sensible and latent energy, whereas thermochemical energy is released or absorbed when a chemical reaction occurs with the heat of a reaction. Sensible and latent heat storage is mostly considered for building applications, although thermochemical energy storage has been gaining a lot of interest recently. In sensible heat TES, heavy materials, such as concrete, stone, etc., are used to store significant amounts of thermal energy. On the other hand, a larger amount of energy per volume can be stored by using the phase transition of the storage materials (PCMs) in latent heat TES [7].

Due to the limited number of sunshine hours in a day, the working of solar systems is also restricted. As soon as the sunlight disappears, the system is deprived of its energy source, thus ceasing its operation at night. This problem can be addressed by integrating a thermal storage system with solar systems. Over the years, latent heat storage (LHS) has been the focus of study for thermal storage [8,9]. The concept of LHS is to incorporate a material that changes phase during its operating temperature range and releases/absorbs a large amount of latent energy, as per the requirement during the non-solar hours.

## 2. Configurations of SPCMS

**Figure 1:**
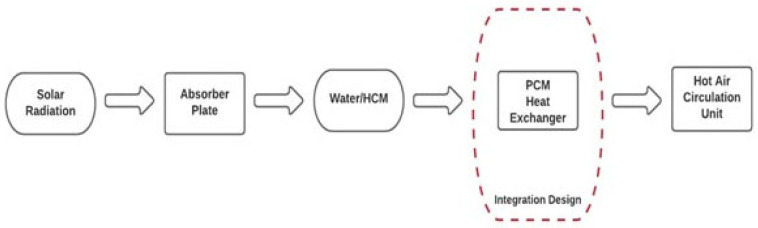
General flow diagram of an SPCMS.

An SPCMS refers to a system that concentrates sunlight on collector plates or drums to maximize the absorption of heat energy from the sun. This heat energy can be subsequently used to heat water or air or be directly used in a building. In PCM solar systems, solar energy is used to charge the PCM during the daytime so that this stored energy can compensate for energy requirements in the absence of sunlight at night. A general flow diagram of an SPCMS is shown in Figure 1.

The basic principle of SPCMSs involves a solar radiation absorber employed to absorb the heat of the sun and, subsequently, transfer this heat to a PCM either directly or through a secondary medium, such as water. This heat transfer causes the PCM to melt down, which is known as charging the PCM. A charged PCM acts as a thermal storage battery. This thermal energy (stored as latent heat) of the PCM is utilized as per need to share the heating and cooling requirements of buildings, and this stage is known as the discharging of the PCM. The PCM-based thermal energy storage systems used in buildings come in two types: active or passive storage systems. Active systems involve auxiliary devices to help charge and discharge a storage tank. On the other hand, passive systems do not need such heat exchangers or forced fluid motion to extract heat or cold from the storage. The incorporation of PCMs into buildings can take two forms: (1) PCMs in building components, like walls, floors, ceilings, etc., and (2) PCMs in heat and cold storage. The former are the passive systems, whereas the latter are active systems [10]. 

For example, a solar PCM system employed for indoor temperature stabilization consists of a flat plate collector, a pump to circulate water in the tubes attached to the bottom side of the collector to heat the incoming water, and a heat exchanger to heat the air coming from inside of a room. Typically, a room having a volume of 50–100 m^3^ requiring a temperature increase up to 25 °C requires a flat plate collector with a surface area of 300–400 m^3^ that is capable of having irradiation in the range of 5–7 kWh/m^2^/day. The flat plate solar collector increases the temperature of the water by 50–90 °C. The PCM phase change temperature for indoor temperature stabilization is typically in the range of 20–100 °C, and its thermal conductivity is generally lower (0.1–0.6 W/m.K). The capacity of the pump to circulate water to the flat plate is normally in the range of 150–300 L.

Many researchers have also investigated the thermal characteristics of active and passive SPCMs simultaneously in a single configuration. Concentrated photovoltaic panels (CPVs) using PCMs were investigated for heating the circulating water through pipes for active and passive system configurations [11]. During the daytime, the panel temperature was reduced due to the melting of PCM, which increased the efficiency of the panels. Subsequently, the charged PCM was used to heat the water at night, thus doubling the advantage of this setup. A two-dimensional model was developed and numerically simulated for the CPV layers with the integrated cooling system. A nanofluid was employed as a heat transfer fluid (HTF) to improve the thermal characteristics of the system. The proposed system was reported to achieve a 60% reduction in the CPV average temperature when compared to the standard PCM-PV and water-cooled systems. Interestingly, the efficiency was increased by 224% after 20 min for the CPV systems. The maximum PCM temperature was maintained below the allowable temperature to avoid its performance deterioration. The use of a nanofluid as the HTF resulted in an increase in the CPV efficiency by 2.7% and a reduction of the PV maximum temperature and the PCM melting time by 4 °C and 12%, respectively. The effect of different arrangements of PCM plates in the water tank was found to be negligible. A comparison of active and passive SPCMSs in the weather conditions of New Zealand [12] demonstrated that an active system is a better option due to its enhanced heat transfer rate and the ability to extract/add heat on demand using control systems with a minimal input of energy. Two identical huts were prepared for the study; the first hut was equipped with wall-impregnated wallboards (passive approach), whereas the other hut was provided with active air-PCM heat storage units. The experimental facility was also equipped with an air-conditioner programmed to switch on at the upper limit of the comfort temperature. It was concluded that the active system prolonged the time taken by the cooling space to reach an upper level by two hours when compared to the corresponding passive system, and the energy difference between the two systems was found to be only 8%. Moreover, the active SPCM configuration was found to consume 22% less energy.

The scope of this review lies in exploring the different design approaches of SPCMS. Any efficient and effective techniques that could be used to improve thermal storage would also be investigated. Since the selection and quantity of a PCM is strongly dependent on the operating environmental conditions, the effect of several key performance factors would be enlightened in this regard, along with the optimization techniques of such systems. Finally, a comparison of the economics of using active and passive SPCMS will be elaborated, along with a critical analysis.

### 2.1. Active Arrangements

SPCMSs requiring auxiliary components for their operation and an external source of energy are referred to as active. This section includes a discussion regarding various design configurations, components/apparatus, and operating mechanisms, as well as the numerical and experimental techniques used to analyze these systems. Moreover, it also includes a thorough overview of various critical performance parameters (melting time, charging profile, etc.) for active SPCMSs. Table 1 contains a comprehensive review in this regard. 

### 2.2. Passive Arrangements

The SPCMSs that do not require any auxiliary component or an external energy source for their operation are referred to as passive solar PCM systems (SPCMS) [22,23]. Passive systems involve the incorporation of PCMs into the walls, roofs, and windows of buildings, and unlike active systems, they do not have any auxiliary setup. Glazed roofs are used to better light a house but, at the same time, this cause thermal discomfort due to the thermal insulation of the glaze. This section includes the discussion of the operating mechanism and analysis techniques to study such systems both numerically and experimentally. Moreover, various areas of study, such as melting time and charging profile, are explored for passive SPCMSs. Table 2 lists a comprehensive review of the research studies for this type of SPCMS. 

### 2.3. Type of PCMs Used in SPCMS

PCMs can be regarded as latent heat storage materials. These materials have higher values in terms of specific heat capacity, implying a high concentration of energy is stored per unit mass due to the phase change process (latent heat). One of the key advantages of using PCMs is that they can be employed in applications having small temperature differences while storing or retrieving energy. These undergo solid-liquid or liquid-gas phase transitions in a working range of temperatures. The volumetric transition during a liquid-gas phase transition is significantly higher than that of a solid-liquid PCM; hence, these are less suitable for building storage applications due to having limited available space. 

**Figure 2:**
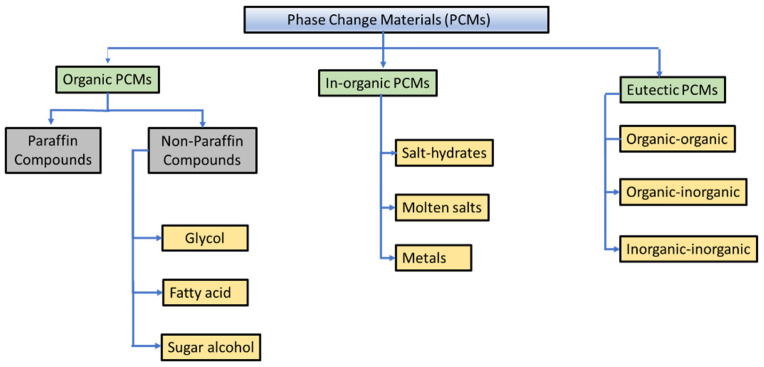
General classification of solid-liquid PCMs.

Figure 2 shows a general classification of PCMs that is employed in solar thermal systems. The solid-liquid PCMs can be further categorized as inorganic, organic, and eutectic PCMs. Examples of organic latent heat storage materials are paraffin, glycol, fatty acids, sugar alcohols, etc., whereas hydrated salts, molten salts, aqueous solutions, water, and metals fall into the category of inorganic PCMs. Finally, eutectic PCMs are a mixture of two or more miscible pure PCM constituents having a single transition temperature. Different PCMs are studied as potential TES materials for temperature reductions within buildings.

PCMs are best suited for thermal storage due to their low cost, easy preparation/availability, high latent heat in terms of fusion, flexible transition temperatures, high storage density, and isothermal operation [13]. The thermal properties of the PCMs commonly used in solar thermal applications are presented in Table 3. Several materials can serve as PCMs, such as paraffin waxes, salt hydrates, fatty acids, and sugar alcohols. Although salt hydrates and fatty acids have relatively higher thermal conductivity, they are chemically active and undergo incongruent melting. Therefore, paraffin waxes are widely used because they are cheap, readily available, and chemically stable [13]. Table 3 shows the thermal properties of commonly used PCMs in solar thermal applications. It can be clearly deduced from Table 3 that the thermal conductivity of commonly used PCMs is quite low. The low thermal conductivity of PCMs, resulting in low thermal performance, has been a major area of research in recent times. 

Different methods to increase the thermal conductivity of PCMSs have been investigated in the past by researchers to address this issue. Bodin et al. [41] experimentally investigated the use of carbon nanostructures in paraffin to enhance its thermal conductivity. Taunite carbon nanotubes were embedded in grade 2 paraffin. It was found that the heat conductance and heat capacity of the nano-modified paraffin were enhanced in comparison to the original paraffin. The use of nano-modified paraffin was also reported to improve the economic feasibility of heat pumps. Shchegolkov et al. [42] performed experiments to investigate the effect of modifying paraffin using multilayer carbon nanotubes with nickel-zinc ferrite. The range of measurements of the temperature field was from 20 to 75 °C. Other methods to improve the thermal conductivity of PCMs include the use of a graphite powder/matrix, internal fins, honeycomb fillers, and carbon fibers [43,44,45,46,47]. The details of these techniques are not in the scope of this review.

## 3. Key Performance Indicators (KPIs) for SPCMSs

SPCMSs have a lot of ground to cover when it comes to their performance. Therefore, comprehensive knowledge of the major critical parameters is indispensable. The subsequent sections discuss the technical and economic indicators responsible for a significant improvement in the performance of SPCMSs.

### 3.1. Technical/Thermophysical Indicators

In order to develop various efficient configurations for SPCMSs, the focus of researchers has shifted towards the optimization of these systems. This initiated a new paradigm towards efficient, low-cost, and clean energy systems. The idea adopted was to investigate the performance of SPCMSs by varying various parameters, such as the tube pitch, tube diameters, the angle of the solar collector, the thermophysical properties of the used PCM, etc. The following is a list of the most important technical and thermophysical performance indicators for SPCMSs.

- Transition temperature of a PCM: The transition temperature of a PCM should be within the operating range of the system in an optimal manner [48]. The pull of heat during charging and the push of heat during discharging should be made equal by ensuring an equivalent ΔT between the phase change temperature and the supply and demand of the HTF. Generally, the applications involving heating requirements employ PCMs that have a phase change temperature in the range of 20–100 °C, except for solar thermal electricity, where a phase change temperature in the range of 400–1000 °C is required. On the other hand, cooling applications usually require a PCM with a phase change temperature in the range of −20–20 °C. PCMs with a phase transition temperature in the range of 21–28 °C are preferred for providing thermal comfort in residential buildings.
- Density of a PCM: A high-density PCM is recommended when storing a higher energy content in a specified volume. In this way, the volume of the thermal storage can be reduced significantly [48]. The density difference between the solid and liquid phases should be kept minimal to avoid ullage, but this will reduce free convective heat transfer, especially during melting. The density of paraffin wax usually lies in the range of 800–900 $kg/m^{3}$.
- Latent heat of fusion of a PCM: A higher value for the latent heat of fusion is preferred. A high latent heat results in more energy storage [49]. The latent heat of fusion for paraffin wax is between 200–300 kJ/kg, whereas for metallic PCMs, its value is found to be in the range of 25–100 kJ/kg.
- Specific heat of a PCM: The specific heat of a PCM should also be high. A higher value for specific heat, again, corresponds to higher-density energy storage, which is desirable [48]. The entire objective of using PCMs is to maximize latent heat transfer while minimizing sensible heat transfer.
- Thermal conductivity: The thermal conductivity of a PCM should be as high as possible. However, the main reason for the limited commercial success of PCMs both in active and passive setups is their inherently low thermal conductivity. Various techniques can be used to increase a PCM’s thermal conductivity, including the use of graphite powder, carbon nanotubes, graphene, honeycomb fillers, aluminum matrices, carbon fibers, nanoparticles, fins, and heat pipes [50]. For instance, the thermal conductivity of paraffin wax was found to increase from 0.21 W/m.K to 4.09 W/m.K when using a composite of paraffin with aluminum powder [50].
- Inter tube distance in a PCM heat exchanger: Heat exchangers with several tubes are recommended to improve the thermal conductivity of SPCMSs. The distance between the tubes of such heat exchangers plays a significant role in overall thermal performance. Intuitively, as the distance between the tubes decreases, the system becomes more compact, resulting in better performance and increasing the area of contact between the HTF and PCM. This compactness not only reduces the loss of heat to the surrounding area but also generates space for more tubes [51].
- Insulation of SPCMSs: The transfer of energy from the PCM to the desired medium occurs with the loss of energy to the surroundings. Minimizing such heat loss will result in the significantly better thermal performance of the system. Polyurethane and wood are commonly used for insulating a SPCMSs [52].
- Circulation flow rate: Useful heat gain is found to increase with the increase in the flow rate of the HTF, which, consequently, causes an increase in heat transfer to the facility in which the system is employed [53]. Normally, HTF flows at a rate of 2–15 L/minute in a SPCMS.
- Cascaded PCM arrangement: Using two PCMs with different transition temperatures results in higher thermal conductivity and, consequently, better performance [54,55]. This technique maintains a consistent ΔT between the HTF and the phase change temperatures of the PCM. An increase of approximately 5% was reported when using cascaded latent heat storage [55]. Usually, no more than three cascaded PCMs are used in an SPCM-based heat exchanger.
- Internal fins: Another archetype for performance improvement comes in the form of an internal fin structure. The use of fins increases thermal conductivity by increasing the effective heat transfer area, thereby increasing the heat transfer rate. Numerical models are used to optimize the number of fins, their diameters, and the length of fins for better performance. Jia et al. [56] found an optimum length-to-radius ratio of 0.75. The authors used a total of six fins and found more than a 50% reduction in discharging time (3600 s using fins compared to 7700 s without using fins).
- PCM layer thickness: PCM layer thickness is an essential indicator of an SPCMS design. PCMs with greater thicknesses tend to increase the overall volume of the system, whereas a small PCM thickness results in performance degradation. Therefore, optimal layer thickness must be carefully selected. Numerical-simulation-based techniques lead to such optimized PCM layer thicknesses [57]. A PCM thickness of 0.01 m was found to produce the best results under the operating conditions of the thermal system under investigation [57].
- Solar irradiation and climatic conditions: Atmospheric conditions are one of the most critical parameters to be considered when designing SPCMSs because several parameters, such as transition temperature, the mass of a PCM, the estimated solar irradiation, etc., are dependent on the climate of a particular place [58].
- Area of the solar collector: Based on climatic predictions and energy storage demand, the area of the installed solar collector is a key parameter to ensure that sufficient solar radiation will be absorbed and thermally stored for use during off-solar times [59,60].

**Figure 3:**
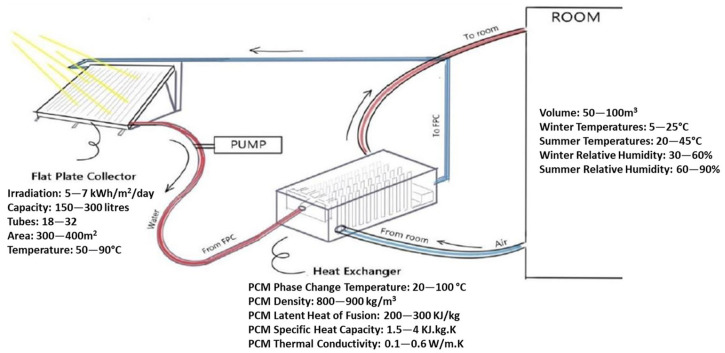
Typical characteristics of indoor temperature stabilization using solar PCMs.

An overview of these typical parameters for an active solar-based PCM system is presented in Figure 3:

The governing equations for the thermal analysis of an SPCMS are

(1) $$\frac{\partial \rho }{\partial t}+\frac{\partial u_{i}}{{\partial x}_{i}}=0$$

(2) $$\frac{\partial \rho u_{i}}{\partial t}+\frac{\partial \rho u_{i}u_{j}}{{\partial x}_{j}}=-\frac{\partial p}{{\partial x}_{i}}+\mu \frac{\partial^{2}u_{i}}{{{\partial x}_{j}}^{2}}+S_{B_{i}}+S_{Mi}$$

(3) $$\frac{\partial \rho h}{\partial t}+\frac{\partial \rho u_{i}h}{{\partial x}_{i}}=\frac{\partial }{{\partial x}_{i}}k\frac{\partial T}{{\partial x}_{i}}$$

where u (in m/s) is taken as fluid velocity, μ (in kg/m.s) represents dynamic viscosity, p (in N/m^2^) denotes the pressure, g (in m/s^2^) represents gravity, Si (in N/m^3^) is taken as the source term, and *ρ* (in kg/m^3^) signifies the density. The buoyancy-forced flow of a PCM liquid is generated due to the melting process, which is an unsteady, incompressible laminar flow. Natural convection produces a density difference for the duration of the melting process because of the gravitational effects. The density discrepancy is estimated by applying Boussinesq’s correlation, which includes thermal expansion coefficient, β, (in *1/K*) temperature difference, density changes ρ, and the buoyancy source term $S_{B_{i}}=\rho \beta \left(T-T_{l}\right)g$. Furthermore, k (W/m.k) signifies the thermal conductivity, T (*K*) represents the temperature, and h (kJ/kg) is the enthalpy in Equations (1)–(3).

In order to efficiently design a system, the aforementioned key indicators and generalized rules are extremely useful. Additionally, the thermal performance indicators that provide a potential design basis for researchers are also extremely valuable. Mazhar et al. [61] carried out an experimental investigation to enhance the performance of a PCM to harness grey water (GW) in domestic buildings. The most important indicator defining the performance of the PCM, known as the Stefan Number, was defined as

(4) $$Stefan\;Number=\frac{Sensible\;heat\;transfer}{Latent\;heat\;trasnfer}=\frac{mC_{p}(T_{final}-T_{initial})}{mL_{f}}$$

where *C_p_* (in kJ/kg.K) is the specific heat at constant pressure, and *L_f_* (in kJ/kg) is the latent heat of melting. A Stefan value equal to 1 indicates equal sensible and latent heat transfer, whereas a value closer to 0 depicts higher latent heat transfer, which is more desirable for an SPCMS. The same researcher also suggested that for the indoor heating/cooling applications of buildings, for an SPCMS, the objective is to minimize the ΔT between the maxima and minima temperatures of the indoor air. Afshan et al. [62] performed an experimental investigation to study the effect of the aspect ratio on the performance of a latent thermal storage unit for solar thermal applications. The experimental setup consisted of three cylindrical storage tanks with different aspect ratios (height to diameter) 320:330 mm for 1:1, 560:250 mm for 2:1, and 720:240 mm for 3:1. PCM balls were placed in the top 50 mm and bottom 50 mm spaces. When the water in the storage tank was stratified, the efficiency of the TES and solar collector system increased. The stratification of the water in tanks was produced by the difference in the density between the hot water and the cold water. The hot water flowed from the top side while the cold water entered from the bottom side. The Richardson number was primarily used to describe the stratification and was estimated by using Equations (5) and (6):

(5) $$Ri=\frac{g\beta H\left[\left(T_{H-in}\right)-\left(T_{Lb}\right)\right]t}{{v^{2}}_{sf}}$$

(6) $$v_{sf}=\frac{V}{\pi r^{2}}$$

Here, *v_sf_* (in *m/s*) is the characteristic velocity of flow. It was concluded that stratification was influenced by the PCM balls in the storage tank irrespective of the aspect ratio used. The increase in the stratification level was found to increase the instantaneous heat transfer rate. The cumulative heat transfer decreased with the passage of time as the aspect ratio increased. The Richardson number also depicted the resistance of the PCM balls, and it increased throughout the charging process, especially in the case of an aspect ratio of 1:1.

### 3.2. Economic Indicators

Researchers are working incessantly on designing economically efficient systems for heating/cooling purposes. There has been a lot of research conducted on PCM-integrated systems that have been proven to be efficient in terms of saving energy. Kong et al. [18] experimentally analyzed a hybrid system of PCM wallboards with excellent thermal and mechanical properties that were integrated with a solar thermal system. The result showed a 44.16% reduction in daily energy consumption. Saafi and Daouas [63] employed a brick wall with PCM impregnated on both surfaces (the inside surface and the outside surface) to investigate the energy-saving potential of the system. The authors came up with the conclusion that the outside surface of the brick wall with the PCM resulted in an energy-efficient system. Several studies conducted on single- and multilayer PCM configurations showed that the multilayered layouts resulted in better energy saving than the single-layer layouts [64,65]. The local climate conditions also affect the energy-saving potential of a PCM-integrated building to some extent, with energy saving ranging from less than 1% [64] to up to 90% [66]. Sovetova et al. [67] assessed the energy efficiency performance of PCM-integrated residential buildings in eight different cities with 13 different PCMs with the help of Energy Plus software, version 7.3. The reduction in energy consumption in the targeted cities fluctuated in a range from 17.97% to 34.26%. Qu et al. [68] studied the influence of several critical parameters on energy savings when using PCM-integrated buildings in the summer season. It was found that a considerable energy-saving rate could be achieved (up to 34.8% in this case) with the proper selection of PCMs in accordance with the climatic conditions. Alam et al. [69] investigated the energy-saving potential of PCMs in eight major Australian cities, which represent six climate zones. The results showed 17–23% annual energy savings when using the system under investigation, depending on the local weather. Wang et al. [70] evaluated the performance of PCM wallboards in air-conditioned buildings during the summer and winter seasons. Different room locations on the middle floor of a building were studied. The results concluded that during the summer season, the east and the west walls have the highest energy-saving rates, and a 27.78% energy-saving rate can be achieved in the east wall model. During the winter season, the south wall has the highest efficiency, which can reach up to a maximum value of 96.2%. The PCM wall was reported to have the fastest payback period of 21.65 years. Zhao et al. [71] studied a solar heating system using a PCM storage tank and compared it with a conventional water tank heating system for comparison in terms of energy saving. The results showed a 34% increase in energy-saving capability. Devaux and Farid [72] studied PCM underfloor heating systems, and the analysis showed great potential in terms of peak load shifting. Overall, 32% and 42% for energy and cost savings were reported, respectively. Calise et al. [73] performed a dynamic simulation for an energy comparison between building-integrated photovoltaic (BIPV) and building-integrated photovoltaic/thermal (BIPV/T) collectors in TRNSYS. A payback period of 4.5 years was reported using the BIPV-based systems. This demonstrates that the BIPV-based system is more energy efficient than the BIPV/T-based systems. Researchers have used different tools to measure the economic impact of PCM solar systems. Calculations regarding energy savings, cost savings, electrical efficiency, payback period, etc., for different configurations of SPCMs have been reported in the literature and are summarized in Table 4.

## 4. Conclusions and Future Recommendations

SPCMSs promise a bright future in terms of providing clean and low-cost energy while enhancing the share of renewable energy in the energy mix of buildings. However, due to the low thermal conductivity of PCMs, SPCMSs have always been at a disadvantage, restricting their applicability, especially in passive configurations. In this spectrum, an encyclopedic review of the novel design concepts of active and passive SPCMSs is presented in this paper. A comprehensive discussion regarding the performance and economics of these systems is also presented, which has the potential to assist designers and researchers in making further improvements. When considering all the matrices of performance, savings, and applicability, it is concluded that active solar systems—despite having some energy-consuming components—provide better energy savings when compared to passive solar systems. The payback time for active SPCMs is usually below 10 years, whereas this is greater for passive configurations. The thermophysical properties of PCMs and the technical design parameters influencing the performance of SPCMSs were also deliberated. The debate of this paper established that SPCMSs have now been improved to such an extent that they can be used practically in domestic buildings to fulfill partial heat/cooling loads when used in combination with the status quo technologies. Nevertheless, these systems have immense ground to cover to compete with commercial electrical storage systems. In this regard, a few future recommendations are listed below:

- The thermal conductivity of PCMs, despite obtaining several improvements through various techniques, is still the main constraint that limits the efficiency of SPCMSs and, thus, needs to be investigated further. Nano-modified PCMs, along with more efficient finned configurations, can be explored further to fully explore their potential for enhancing thermal characteristics.
- Research should be conducted to a) increase the density of PCMs and b) decrease the volume of the system and, thus, increase energy storage for compact domestic applications linked with solar thermal energy.
- The configuration and geometry of tubes exchanging heat with PCMs need to be explored further so that complete charging and discharging can be achieved within a shorter timespan without depositing hard water sediments over the long term, as has been observed in most studies.
- Substantial energy savings can be achieved in buildings with PCM incorporation, as has been shown in this review. However, it is imperative to take great care when selecting a PCM for a particular application by considering their phase change temperature, thermal stability, and compatibility. Most importantly, it is vital to ensure the setup is not toxic or corrosive, as this would be in close contact with humans.
- Due to unpredictable weather fluctuations, these systems should be tested under real conditions in pilot studies instead of simulated conditions for more accurate results.
- Focused research on the prevention of energy losses should be performed, especially considering usage with fluctuating solar water temperatures.
- The freezing of solar water at night under rare conditions, in which the temperatures are below subzero, is a major research gap that must be further investigated to mitigate operational risks.

## Figures and Tables

**Table 1:** Summary of research into the active domain, characterized by numerical and experimental studies.

Subject	Overview	Outcome	References
PCM storage for solar DHW	▪ The system comprised of a solar collector, a pump for forced circulation of water, a water tank, circular PCM tubes and an auxiliary heater. Lumped capacitance assumption was used due to use of a composite PCM having high thermal conductivity. The results of both configurations, with and without PCM were compared. Solar collectors were used to heat the water which in turn transferred thermal energy to composite paraffin PCM present in circular tubes in the water tank. Energy stored in PCM prevented auxiliary heater from switching on and saves electricity consumption at night by releasing its energy to incoming cold water [13]. ▪ The PCM was placed in the HTF solar loop from the solar DHW system couple with flat plate collectors. A back-up system comprising of electric resistance was placed in the top quarter of water tank to meet energy requirements in the absence of solar radiations at night. This configuration was numerically studied under different weather conditions (summer and winters conditions) and by changing critical system parameters. System optimization using a novel genetic algorithm is proposed, for the system configuration analyzed [14].	▪ Annual solar energy delivered to the consumer increased from 6457 MJ without PCM to 6692 MJ With PCM. Annual electricity backup for auxiliary heater reduced for PCM incorporated configuration from 843 MJ to 723 MJ. Collector efficiency using PCM increased from 45.2% to 46.5% [13]. It was concluded that although the PCM embedded system provided more energy, reduced electrical backup and greater collector efficiency, the effect of incorporation PCM is not significant [13]. The maximum solar fraction was found to be around 84% which corresponds to minimum energy of 40 KWh delivered by the backup heating system. The inclusion of PCM module was found to induce significant increase in solar fraction and resulted in significant reduction in the effective volume of the storage tank [15]. The proposed system was suggested to have further optimization based on yearlong simulations, different end user profiles and various system regulation parameters.	[13,14]
DP-SAH equipped with capsules of PCM	▪ This research was concerned with the numerical and experimental investigation of the thermal performance of a double-pass solar air heater (DP-SAH) using paraffin wax capsules integrated with a finned absorber plate. The major objective was to study the effect of solar irradiance and air flow rate on the performance of the DP-SAH by utilizing latent thermal storage. ▪ The apparatus consisted of a DP-SAH, a glaze cover, a finned absorber plate, a fan, an artificial solar simulator, sensors for measuring flow rate, k-type thermocouples, and solar intensity measurement sensors. The solar collector was embedded with rectangular matrices of a PCM, and the air was passed through both sides of the collector. ▪ Energy balance was employed to numerically calculate the useful heat gain and the heat loss from the top side of the collector plate, and three-dimensional simulations were conducted to investigate the effect of forced convection on the thermal characteristics of the system. The adequacy of the grid was determined by using a mesh-independence study.	▪ The numerical results were found to be in good agreement with the experimental results, and the maximum error between the numerical results and the experimental results was found to be around 15%. ▪ The study showed that an increased air-flow rate resulted in a delay in melting time and a reduction in the melting temperature of the PCM. It was also demonstrated that freezing time has an inverse relation with flow rate. It was also claimed that such a system can also be used at night. ▪ It was observed that the optimal discharging period and the rise in air temperature of the heater were achieved in a reasonable time frame (max. 3 h), implying its feasibility at nighttime.	[15]
Solar double-slope PCM glazed roof	▪ In this study, a system having glazed roofs was constructed for better lighting. However, due to poor insulation, the glazed roofs were found to result in thermal discomfort. The solution proposed for this issue was to embed the double-sloped glazed roof with and without a PCM sandwiched in between the roof layers. The performance of both concepts was then analyzed to draw conclusions regarding their thermal performance. ▪ Furthermore, the effects of changing the various critical parameters of the PCM and glazing, such as roof angle, PCM layer thickness, PCM melting temperature, PCM latent heat, and PCM melting temperature, were studied in Wuhan weather conditions. Air was passed on both sides using fans, and this heated air was subsequently circulated in a room to achieve a thermal comfort zone. ▪ For analytical modeling, the assumptions of negligible radiation heat transfer, uniform material properties, homogenous glazing, and the PCM and optical properties independent of wavelength, were taken. ▪ A heat diffusion equation and Fourier’s law were used to obtain an equation of heat conduction. A simplified equation was derived from this conduction equation by assuming one-dimensional heat transfer and neglecting internal energy generation. On the other hand, A numerical model was also proposed using the ANSYS FLUENT module, which simulated the three-dimensional transient flow of the actual system.	▪ The average error between the experimental data and numerical results was reported to be less than 7%. ▪ The configuration with the PCM-sandwiched double-glazed roof was found to provide better thermal performance when compared to the hollow double-glazed roof configuration. ▪ It was also demonstrated that an increase in the layer thickness of both the glazing and PCM resulted in better thermal performance. Moreover, the change in phase-change-layer thickness of the double-slope PCM glazed roof was more significant for improving the roof heat transfer performance than the change in glazing-layer thickness. ▪ Under the climatic conditions of Wuhan, the phase change temperature between 26–30 °C provided optimum thermal performance. ▪ Thermal performance was reported to also increase by choosing a PCM with higher latent heat and by decreasing the tilt angle of the roof glazing.	[16]
Active-slab-containing PCM integrated into solar air collector	▪ An experimental study of a horizontal concrete slab of a building, having macro encapsulated PCM channels fitted inside, was conducted to investigate any possible reduction in the energy requirements of the building. The main reason for the installation of the PCM within the slab was to eliminate the energy losses to the environment. ▪ The model was constructed to be tested under real-time Spanish weather conditions (severe and mild). The apparatus consisted of a small cubical building having a horizontal concrete slab with macro encapsulated PCM channels. The slab had a passage of air circulation to melt the PCM effectively by using the air warmed through a solar air heater. ▪ A control system was employed to change the melting rate of the PCM by using the flow of warm air coming from the solar air heater. The performance of this novel system was compared with a conventional heating system. ▪ Three different operating programs were investigated in this study: heat storage (HS), day discharge (DD), and day discharge and set point (DD + SP).	▪ For several of the cases considered, incomplete melting was observed, which suggested further detailed investigation regarding the ratio of the size of the solar air heater and the quantity of PCM. ▪ The complete melting of the PCM showed energy savings of 55%, whereas in the case of partial melting, an energy saving of 20% was reported for the HS configuration. ▪ The optimization of the control system algorithm was reported to increase the energy savings. ▪ For the DD experiments, a 25 % energy saving (on average) was reported under severe weather conditions, and 40% energy savings were achieved for mild weather conditions. ▪ The difference in the results between the DD and DD+SP arrangements was found to be negligible.	[17]
Preheating of ventilation air using a solar PCM	▪ The objective of this numerical and experimental study was to investigate the thermal comfort stabilization of a room during ventilation at a minimum cost. It was proposed that preheated ventilation air must be supplied using the solar heat stored beneath a ventilation window in a PCM. ▪ The nonlinear thermal properties of the PCM, heat transfer model, and buoyancy-driven laminar flow models were integrated to numerically simulate the PCM air solar heat exchanger. ▪ The apparatus consisted of one full-scale hot room and one full-scale cold room. The cold room maintained its temperature through a cooling coil. A PCM heat exchanger was placed in between the hot and cold rooms, and the heat exchanger consisted of PCM plates closely spaced with air in between them. ▪ For the experimental setup, the hot room was ventilated through the air from the cold room. The cold room air was made to pass through the PCM heat exchanger, which was being charged using an artificial sun, and it was heated in the process. ▪ The air entered from the bottom side of the heat exchanger, in between the PCM plates, and subsequently entered the hot room through the ventilation cavity of a double-glazed ventilation window. ▪ Then, the air was made to pass through the heat exchanger only when the solar radiation decreased below 200 W/m^2^. In the case of solar radiation having a higher value, the air was flown directly into the room because it was sufficiently heated by the sun itself, thereby eliminat-ing the requirement of preheating. ▪ A three-dimensional model was simulated using COMSOL Multiphysics by considering the PCM heat transfer as a conduction heat transfer. The effect of convection through the air was included by a convection coefficient, h, which was evaluated through empirical correlations for the Nusselt number. The effect of the buoyancy effect in the fluid flow was also incorporated.	▪ A close agreement between the numerical and the experimental results was found, which rationalized the use of this numerical model for the further optimization of the PCM air solar system. The error between the experimental and numerical studies was found to be less than 6%. ▪ The heat released through a fully charged PCM was 93.31 MJ/m^3^. The system was exposed to 550 W solar charging for 6 h, which is equivalent to the average daily radiation in Denmark in April. ▪ The authors studied the relationship between various depths of PCM tubes, as well as the gap between the tubes, with the charging and discharging time of PCM. ▪ The maximum melting, having a 90.2% melting fraction for a PCM with a 90 mm PCM plate, was reported, and a melting fraction of 59.5% and 69.8% were found for 100 mm and 110 mm plates, respectively. ▪ It was observed that the depth of the PCM plate did not affect the discharge time. Nonetheless, with a 90 mm plate depth, the energy released was 93.31 MJ/m^3^, and the optimum air gap was found to be 6 mm. This configuration can be employed for both summer cool-ing and winter solar energy storage applications. ▪ The discharge rate was found to decrease gradually with an increase in the gap between the plates. This slow reduction indicated that the energy released was a dominant factor in the total solar energy absorbed by the PCM.	[1]
Hybrid PCM system using an active composite wall	▪ The system comprised a passive PCM wallboard linked with an active solar thermal system, constituting a hybrid system. In this study, an expanded perlite-based composite was used as the PCM, and this was integrated into a wallboard; it was subsequently incorporated into a solar thermal system using capillaries. ▪ The two PCM wallboards were prepared using newly made PCM particles, styrene acrylic emulsion, environmentally friendly paint, and fiber glass. Wallboard-I was plain, whereas wallboard-II had grooves for the integration of the capillaries. Additional aluminum powder was used in wallboard-II to enhance its thermal conductivity. ▪ The two same-sized rooms, a PCM room and a reference room, were prepared to conduct three distinct sets of experiments. Both rooms comprised a heat exchange tank connected to the solar collectors.	▪ The incorporation of PCM wallboards into a solar thermal system produced satisfactory results. ▪ Thermal conductivity was found to increase by 109% with the addition of aluminum powder in wallboard- II. ▪ The supply water temperatures for the PCM room and reference room were found to be almost the same, which demonstrated that both supplies got almost the same amount of solar thermal energy. Moreover, the return water temperature of the PCM room was lower than the return temperature of the reference room, implying a larger heat transfer temperature difference for the PCM wallboard system compared with that of the reference room. ▪ The PCM wallboard had a maximum pressure endurance of 98 N and a maximum flexure strength of 0.41 MPa, with a 7.85 mm deflection, which indicates high wall strength. ▪ Daily energy consumption was reduced by 44.16% using this hybrid system. A payback time of 3.32 years was estimated for the proposed configuration.	[18]
PCM-integrated solar chimney	▪ The main objective of this experimental study was to investigate the effects of a PCM on the performance of two different solar chimney prototypes. ▪ The first prototype analyzed the behavior of Rubitherm (RT44) PCM panels on a simple-build solar chimney. The second prototype was a modified version of the first type of build according to the design of SPA (Solar Platform of Almeria). ▪ The experiments were conducted with and without the PCM, comprising a 6 h long process going through seven process phases. The cycle started with (1) initialization, (2) the charging of the PCM, (3) air circulation for maintaining a constant source of heat, and (4) the subsequent removal of the air source. In the last three phases, the study repeated phases 1, 3, and 4. A high-fidelity instrumentation system was used to measure several critical parameters, like air temperature, surface temperature, mass flow rate, etc.	▪ The desired objectives of the experimental study were obtained, despite the incomplete fusion of the materials within the panels. ▪ An additional layer of insulation behind the plywood layer helped avoid heat loss across the chimney and PCM panels, providing thermal inertia. ▪ The overall performance of the system was found to be a strong function of the thermal characteristics of the chimney walls. After a charging period of 6 h, an average ventilation rate of above 70 m^3^/h could be obtained with a low gain of 550 W/m^2^ provided by a series of seven halogen lamps. ▪ During the discharging period of 6 h, the incorporation of the PCM into the solar chimney provided a slower decrease in the ventilation rate, with an overall higher ventilation rate. ▪ The solar chimney equipped with the PCM was reported to provide a 33% higher temperature when compared to the case with no PCM. ▪ The integration of the PCM panels was found to enhance system stabilization over a large range of operating conditions.	[19]
Dual-air-channel PCM system with solar wall	▪ The multi-functional dual-air-channel solar wall scheme with a PCM consisted of a glass cover, an absorbing plate, several PV cells, a pump, a cycle pump, a water storage tank, PCMs, an insulation layer, a wall, and the vents. The PV cells were laminated on the front surface of the absorber plate, with a coverage of 60%. ▪ Over an entire year, the system was designed to meet the different seasonal energy and thermal comfort requirements of the building. Therefore, it was tested for three seasonal modes: (1) the winter mode, (2) the summer mode, and (3) the transition mode (spring and autumn). ▪ In the winter mode, heat was transferred between the absorber plate and the air via the convection heat transfer mode during the daytime, and the heated air was used to charge the PCM. At nighttime, the heat stored in the PCM was subsequently utilized to maintain the thermal comfort level of the system. ▪ In the summer mode, a pump was used to circulate water during the daytime, which gained heat from the absorber plate. The heat of the air was absorbed by the PCM, and it was subsequently cooled by the air at night. ▪ In the transition season mode, the daytime operation was the same for the summer mode, and the nighttime process for the winter mode was employed. ▪ A mathematical model of the entire system comprising of a glass cover, an absorber plate, a PV panel, a water pipe, a tank, airflows in the two channels, an insulation layer, a PCM, a south wall, and room air was formulated for validation. The heat diffusion equation was numerically solved for the glass fiber.	▪ In each working mode, the system exhibited very good electrical and thermal performance, and continuous indoor space heating was improved during the nighttime. The accuracy of the established model was found to be acceptable through model validation. ▪ It was suggested that the performance of the system could be improved with a proper increase in the PV cell coverage and channel height. The electrical power and thermal efficiency during the winter, autumn, and summer experimental days were found to be 2.28 kWh, 1.77 kWh, and 0.94 kWh and 11.75%, 11.45%, and 8.88%, respectively. ▪ The appropriate transition temperature range of the PCM used was found to be in the range of 19–21 °C in the winter days for a maximum PCM temperature value of 5.7681 MJ and 22–24 °C in the transition season days for a maximum PCM temperature value of 4.6735 MJ. This was claimed to provide better continuous space heating performance during nocturnal hours.	[20]
Solar-aided PCM-based space heating	▪ The main goal of this experimental and numerical study was to investigate the thermal performance of a solar-aided latent heat source store for space heating using a heat pump. ▪ The experimental setup consisted of a flat plate solar collector, a thermal energy storage tank filled with an encapsulated PCM, a heat pump with a water-sourced evaporator, an air-cooled condenser, and a room for heating. ▪ Two modes of operation were investigated. The first mode comprised water receiving solar heat from the solar flat plate collector. This water was subsequently used to partly provide heat to the storage tank and, partly, it was used by the water-sourced evaporator of the heat pump as a heat source. The water coming out of the evaporator was sent back to the solar collector. ▪ At night-time, during the absence of solar radiation, the cold water from the evaporator was transported to the storage tank where the heat was extracted, and it started to be evaporated for the heat source.	▪ The numerical model developed, which was based on an enthalpy-based finite difference formulation, for the charging process of the PCM was found to be accurate. ▪ The thickness of the pipes was suggested to be kept minimal in order to minimize the energy storage in the walls of the pipe. ▪ At the start of the process, the outlet temperature of the tank was low, implying that a pipe shorter in length and a tank low in height was preferable. ▪ It was concluded that the thickness of the pipe walls must be thin to minimize the energy stored in the pipe walls. ▪ It was recommended that a solar-assisted heat pump integrated with an air-sourced evaporator could improve the thermal characteristics of the system significantly.	[21]

**Table 2:** Summary of research into the passive domain, characterized by numerical and experimental studies.

Subject	Overview	Outcome	Reference
PCM in gypsum Boards	▪ A gypsum plaster and a salt mixture were two materials used for the experimental investigation into their impact on room temperature reduction, with the data verified, subsequently, by a mathematical model [24]. Two similar experimental chambers were prepared; the walls of the first chamber were covered with ordinary plasterboard, whereas the walls of the second chamber were covered with PCM wallboard. The material for the chamber walls was changed between different compositions of gypsum plasterboards and mineral wool. ▪ The thermal characteristics of a heat storage gypsum-cement (G/C) board were experimentally analyzed. Moreover, a dynamic heat transfer analysis was performed to evaluate the time-lag effect and thermal efficiency of the developed heat storage G/C board with PCMs [25]. The two PCMS used in this research were n-octadecane and Beeswax. ▪ The system consisted of a PCM-impregnated gypsum board, and an analysis was performed to analyze its dynamic thermal performance [26]. Both numerical and experimental investigations were performed. Different experimental techniques, such as differential scanning calorimeter (DCS) and dynamic heat flowmeter apparatus (DHFMA), were employed to characterize the thermal performance of the PCMs	▪ A reduction in the peak temperature of up to 4 K was reported in the test chamber (with walls made of PCM plasterboard) when compared to the test chamber without PCM. A mathematical model was calibrated using experimental data [24]. It was recommended that the proper discharging of the PCM at night is necessary for the maintenance of PCM’s thermal characteristics over a long period of time. ▪ The PCM-containing heat storage G/C boards exhibited a time-lag effect. The use of such heat storage G/C boards can be employed to reduce the energy consumption of buildings [25]. The heat storage G/C boards showed around a 150% increase in thermal conductivity when using a modified PCM material compared to their original PCM. The n-octane PCM was found to have a larger energy reduction effect due to its more suitable phase change temperature. ▪ Negligible subcooling and hysteresis were observed for the PCM-impregnated gypsum board [26]. The results of DCS were found to be similar to those of DHFMA in terms of the heat capacity profiles and the amount of subcooling of the PCMs employed.	[24,25,26]
PCM in Concrete	▪ Clastic light shale ceramsite (CLSC) was employed to absorb paraffin to prepare a PCM-CLSC aggregate. Three PCM concrete thermal storage blocks with different PCM weight percentages (0, 2, 4, and 6) wt% were experimentally investigated. An active thermal storage and release system was used to improve the PCM charging/discharging characteristics [27]. ▪ Experimental investigations were carried out in the Dutch environment using extensive data collection techniques to study the performance of the micro-encapsulated PCM concrete setup. The system comprised four testing chambers; two chambers were comprised of PCM concrete floors and two had normal concrete floors. The test chambers were fully insulated, and heat transfer was achieved through a window located on the south side of the chambers [28]. The latent heat capacity of the PCMs used was 110 kJ/kg, with negligible specific heat capacity. On the other hand, the normal concrete had a negligible latent heat capacity and a 3.3 kJ/kg specific heat capacity.	▪ The thermal conductivity and compressive strength of the blocks decreased with the increase in PCM weight percentage, and the average specific heat capacity increased by 12.54% (2 wt% PCM), 31.60 (4 wt% PCM) and 41.23% (6 wt% PCM), respectively. It was found that heat storage and heat release capacity could be enhanced by increasing the PCM weight percentage, HTF flow rate, and inlet temperature [27]. Furthermore, it was found that CLS could be used to absorb paraffin at an absorption rate of 25.8%. ▪ The results indicated that temperature fluctuation could be minimized by using this setup. This conclusion was made due to the maximum floor temperature being reduced by approximately 16% and the minimum temperature was increased by almost 10% [28]. ▪ However, since the PCM floor was directly exposed to solar irradiation, it is difficult to implement this setup in practice because the floor is always covered with tiles or wood, thus resulting in minimal direct contact between solar irradiation and the PCM. ▪ Further research in this area should be focused on using PCMs with high thermal conductivity and a lower transition temperature.	[27,28]
PCM in Bricks	▪ This parametric study was carried out in Moroccan conditions to numerically investigate the potential of a PCM as a passive solution employed in bricks to reduce the time delay in internal as well as external thermal conditions and to study the thermal stabilization characteristics. ▪ The setup comprised two walls of clay, one with a micro-encapsulated PCM and the other without a PCM. A climatic chamber was used to test the walls, with one side having an external boundary condition and the other with a free-floating boundary condition. The PCM used was RT18, with a melting temperature of 18 °C. ANSYS Fluent software was used to solve the conservation equations with an enthalpy porosity-based model using a finite volume approach.	▪ A time delay of 3 h could be achieved by using the brick wall with the PCM when compared to the conventional brick wall. ▪ The amplitude of thermal response decreased from 10 °C for a normal wall to 5 °C for the PCM-equipped wall. ▪ The latent heat and melting temperature of the PCM were found to be the most critical parameters in this study. Moreover, an improved thermal performance was reported for the cases with a higher concentration of PCM. ▪ PCM integration into hollow brick resulted in higher energy storage and better insulation characteristics. ▪ Conducting studies on the mechanical, economic, and environmental aspects to determine the complete feasibility of such systems was recommended.	[29]
PCM based Free Cooling	▪ An experimental investigation was performed to study the potential of PCM-based free cooling, i.e., storing energy at nighttime in a flat, modular-type heat exchanger incorporated with PCM, using the same energy for cooling in the daytime. ▪ The system consisted of a cabin with an area 64 *ft*^2^. The thermal characteristics during the charging phase and the discharging phase were investigated. The heat exchanger was made up of 10 rectangular stainless-steel panels. A total of 76 kg of HS 29 PCM, with a transition temperature of 28–29 °C, was used. ▪ The system was designed for a free cooling capacity of 0.5 kW and a storage capacity of 15000 kJ. The fan capacity was selected to provide a mass flow rate to charge 76 kg of PCM (HS-29) in 5 h. A data acquisition system was employed to record temperature readings at six different locations in the cabin.	▪ The results revealed that the temperature could be reduced by approximately 2.5 °C in the cabin. ▪ During the initial stage of the charging phase, an instantaneous heat transfer rate of 0.35 kW was reported, and it was subsequently reduced to 0.2 kW after 500 min. ▪ The driving potential for heat transfer, i.e., the temperature difference between the PCM and ambient air, was the major critical parameter. ▪ It was suggested that the efficacy of free cooling can be enhanced by reducing the losses. Moreover, this technology has the potential to replace conventional air conditioners, providing that the incorporation of evaporative cooling is used in this technique.	[30]
PCM Trombe wall	▪ These studies were conducted on a PCM Trombe wall to compare its thermal performance with a conventional concrete wall. An experimental setup studied the heat transfer characteristics in Chinese weather conditions of a Trombe wall using a PCM made of a mixture of 55% decanoic acid and 45% lauric acid. The PCM was encapsulated in a stainless-steel container. ▪ A dual-layer PCM-integrated Trombe wall was numerically investigated using TRNSYS software for Wuhan weather conditions in summer and winter [31]. ▪ The Trombe wall consisted of glass glazing and a brick wall with an air gap of 4–5 inches in between, and this was investigated. The air gap trapped heat during the daytime, which was subsequently transferred into the room through the wall at night [32]. ▪ A PCM was used in the walls for a better heat-storage medium. Paraffin with mixed metal shavings was used for increased overall conductivity and efficiency. The main objective of this study was to achieve walls with a low mass and high thermal efficiency.	▪ Only the PCM in the upper region was melted in the two cases investigated, implying heat transfer to be a two-dimensional phenomenon in such a scenario. A temperature difference of 10 K was reported in the height direction for the Trombe wall, which became double for a higher heat flux case, implying strong temperature dependence. The air temperature rise in the air gap was almost 20 °C [33]. ▪ The room temperature in the PCM wall was less than that of the reference room in summer conditions, whereas it was the same for both walls in winter conditions. The PCM wall exhibited better thermal stabilization characteristics in comparison to the normal wall. The optimal phase change temperature of the external PCM was 30 °C, whereas it was 18 °C for the internal PCM [31]. ▪ It was concluded that the thermal resistance of the solar thermal wall should be as low as possible to maximize solar absorption [32]. ▪ In comparison with the concrete wall, the wall containing paraffin-metal mixtures offered a 90% reduction in storage mass and a 20% increase in thermal efficiency.	[31,32,33]
PCM Shutter	▪ The main objective of this experimental study for two side-by-side compartments (the first one being the reference compartment without a PCM and the other one having a PCM) was to increase the heat transfer rate for shutters placed outside of windows in order to warm a cold room at nighttime using thermal storage. ▪ The thermal performance of a test cell with external dimensions of 7 m × 2.35 m × 2.58 m and having an internal floor area of 5.17 m^2^ was studied (a) with a PCM and (b) without a PCM. CG lauric acid, with a melting point of 49 °C was used as a storage material.	▪ A thermal amplitude of 9.5 °C for the outer air was found, and the maximum radiation collected was 310 W/m^2^. The heat storing capacity of the test volume, when used at night, was found to increase by 4 °C for 4–5 h due to the presence of the PCM. ▪ Although the difference in maximum temperature between the PCM configuration and the reference was in the range of 30% to 40%, the potential improvement due to PCM integration in terms of the minimum indoor temperature was found to be negligible due to large thermal bridge losses and large glazing areas. It was, therefore, recommended to optimize the PCM melting temperature according to mean weather conditions.	[34]
PCM after Mosaic Tiles	▪ A numerical investigation was performed for Hong Kong weather conditions to optimize the thermal characteristics for cooling a residential flat using a PCM-integrated facade oriented at different angles. ▪ A panel of PCM was placed after a layer of mosaic tiles, and cement that was 0.005 m in thickness (between a 0.01 m thick cement layer and 0.1 m thick heavy concreter layer) was used. The melting point of PCM used was 21.7 °C. ▪ Energy-Plus software based on implicit finite difference formulation was used for numerical analysis by using the Gauss-Seidel iterative method. The enthalpy and the specific heat transfer coefficients were corrected upon each iteration.	▪ The building configurations with PCM-integrated facades facing west and east performed better with respect to a decrease in interior surface temperature. ▪ The annual increase in the cooling energy for the system was found to be negligible for the PCM-integrated walls when compared to the base case. ▪ The PCM-integrated building facades were found to be economically infeasible for Hong Kong weather conditions, mainly due to the extensive initial cost of the PCM wallboard. ▪ It was recommended, in the paper, to use a PCM with a higher melting temperature (e.g., 28–30 °C) to increase the energy being released during the PCM solidification phase.	[35]
Air-based Heating System	▪ The performance of an air-based solar heating system was numerically studied for Japanese weather conditions using inhouse thermal load calculation software (i.e., ExTLA, Excel-based Thermal Load Analysis). The main objective of this research was to study the effects of the latent heat and melting temperature of a PCM on an air-based solar heating system. ▪ A simulation was developed to understand the annual thermal load performance of an air-based solar heating system. The models of the solar collector, hot water tank, and thermal storage were validated against the experimental results.	▪ The effect of changing the glass collector plate was found to be negligible because the advantage of low heat loss due to double-glazed glass was nullified by low solar radiation transmittance. ▪ A decrease of approximately 17.9% was found (over conventional heating systems with no thermal storage) for the case with higher underfloor temperatures using additional thermal storage. The reduction in annual load was largely independent of the insulation in the foundation concreter, and this forced the circulation of the air to release higher thermal energy from the thermal storage body.	[36]
PCM in Ceilings	▪ An experiment was carried out for Montreal weather conditions using sun reflectors to direct the solar energy onto a ceiling void integrated with PCM panels for a space heating system (0% of the incident irradiation was reflected onto the PCM panels). The system had a large area for heat storage without needing large volumes for the storage medium, which would be required for sensible heat storage. The main objective was to prevent heat loss [37]. ▪ A comprehensive review of radiant chilled ceilings (RCCs) was presented, with the main focus being on the critical parameters affecting the performance of such systems. It further illustrated the potential of RCCs in terms of energy savings, peak loading shifting, and thermal comfort [38].	▪ This system claimed to have the potential to recover 17–36% of heat lost, which occurred during the initial phase [37]. However, in terms of a quantitative comparison, the efficacy of the system was found to be insignificant. ▪ It was suggested that a PCM with high thermal conductivity and a high phase change temperature would improve the efficiency of PCM-RCC systems. The emphasis was put on the correct quantity of PCM used in buildings, as calculated based on the daily heat gains required for a sufficient cooling capacity. Moreover, system performance degradation was thought to be possible in the case where the PCM was not fully regenerated before the operation [38].	[37,38]
PCM in capasules	▪ A numerical investigation was conducted to investigate the thermal characteristics of a novel staggered configuration of a PCM capsule (n-Octadecane). The effect of the position of the HTF inner tube and its wall temperature on temperature distribution and melting time was analyzed in terms of the Stefan number by using a 2D model simulated using Comsol-Multiphysics [39]. ▪ A rectangular capsule filled with PCM (Rubitherm GmbH, RT_27) was numerically investigated using a finite element method-based solution to observe the effects of mid-separating fins at different inclination angles (0°, 45°, and 90°) [40].	▪ The numerical analysis clearly showed an improvement in heat transfer characteristics and melting rates with the downward motion of the inner tube and with an increase in the Stefan number. The best configuration resulted in an increase of 28% for the downward position of the HTF tube, having Stefan number of 0.15 [39]. ▪ The heat transfer and the storing energy relative to the melting rate were found to increase with the incorporation of a fin inside the PCM capsule. An inclination angle of 0 degrees was found to give the best performance in terms of melting time reduction (20.52%) and an increase in the thermal energy storage rate (36.88%) [40].	[39,40]

**Table 3:** Thermal properties of PCMs.

PCM	Melting Temperature (°C)	Thermal Conductivity (W/m K)	Heat of Fusion (kJ/kg)	Density (kg/m^3^)
Polyglycol E400	8	0.187	99.6	1125
Paraffin C_15_	10	-	205	-
n-Pentadecane	10	-	193.9	770
Caprylic acid (fatty acid)	16	0.149	148.5	901
Paraffin C_16_	16.7	-	237.1	-
Glycerin (organic)	17.9	0.143	198.7	-
n-Heptadecane (organic)	19	0.21	240	760
OM 21	22	0.14	174	891
HS 22	23	0.56	167.6	1540
HS 24	26	0.55	-	1510
Lactic acid	26	-	184	-
n-Octadecane	28	0.148	244	774
HS 29	29	0.382	190	1530
OM 32	33	0.145	157	870
Capric acid	32	0.153	152.7	878
Lauric acid	42	0.147	178	870
Paraffin C_20_	36.7	-	246	-
OM 35	35	0.16	171	870

**Table 4:** Overview of typical energy and economic savings.

PCM	Site	Energy Savings (kW/Year)	Payback Period (Year)	Reference
BioPCM@ ™ M91	Nicosia	20.567	14.5	[74]
PCM27	Hong Kong	3798.34	30.09	[75]
PCM23	Australia	4833.33	-	[75]
PCM29	Iran	2969.65	42	[76]
PCM-enhanced insulation	Miami	19,954	7	[77]
TIM-PCM	Paris	668.8	22	[78]
n-hexadecane	Seoul	326.36	6.88	[79]
n-heptadecane	Seoul	312.18	6.80	[79]
n-octadecane	Seoul	205.37	8.38	[79]
25# Paraffin	China	-	3.32	[18]
Dupon Energain	Aveiro	-	41	[80]
BioPCM^®^ M51	Aveiro	-	18	[80]
BioPCM^®^ M91	Aveiro	-	26	[80]

## Data Availability

Not applicable.

## Abbreviations and Nomenclature

SPCMS	Solar PCM system
PCM	Phase change material
TES	Thermal energy storage
HVAC	Heating, ventilation, and air-conditioning
PTTS	Passive thermal storage system
DP-SAH	Double-pass solar air heater
CPV	Concentrated photovoltaic
HTF	Heat transfer fluid
DHW	Domestic hot water
$S_{i}$	Source term
ρ	Density (kgm^−3^)
β	Thermal expansion coefficient (K^−1^)
T	Temperature (K)
k	Thermal conductivity (Wm^−1^ K ^−1^)
h	Enthalpy (Jkg^−1^)
C_p_	Specific heat (Jkg^−1^K^−1^)
$L_{f}$	Latent heat of fusion (kJkg^−1^)
$S_{Mi}$	Source term for momentum
t	Time (min, second)

## References

[1] Hu Y., Heiselberg P.K., Johra H., Guo R. (2019). Experimental and numerical study of a PCM solar air heat exchanger and its ventilation preheating effectiveness. Renew. Energy.

[2] Zhao J., Ji Y., Yuan Y., Zhang Z., Lu J. (2017). Seven Operation Modes and Simulation Models of Solar Heating System with PCM Storage Tank. Energies.

[3] Jamar A., Majid Z., Azmi W., Norhafana M., Razak A. (2016). A review of water heating system for solar energy applications. Int. Commun. Heat Mass Transf..

[4] Selvaraj D.A., Victor K. (2021). Design and Performance of Solar PV Integrated Domestic Vapor Absorption Refrigeration System. Int. J. Photoenergy.

[5] Xu H., Sze J.Y., Romagnoli A., Py X. (2017). Selection of Phase Change Material for Thermal Energy Storage in Solar Air Conditioning Systems. Energy Procedia.

[6] Yang Y.K., Kim M.Y., Chung M.H., Park J.C. (2019). PCM cool roof systems for mitigating urban heat island—An experimental and numerical analysis. Energy Build..

[7] Souayfane F., Fardoun F., Biwole P.-H. (2016). Phase change materials (PCM) for cooling applications in buildings: A review. Energy Build..

[8] Mettawee E.-B.S., Assassa G.M. (2006). Experimental study of a compact PCM solar collector. Energy.

[9] Iten D.C. (2015). Air-Multiple PCMs for the Free Cooling and Ventilation of Buildings Air-Multiple PCMs for the Free Cooling and Ventilation of Buildings. Ph.D. Thesis.

[10] Meng E., Cai R., Sun Z., Yang J., Wang J. (2021). Experimental study of the passive and active performance of real-scale composite PCM room in winter. Appl. Therm. Eng..

[11] Nasef H., Nada S., Hassan H. (2019). Integrative passive and active cooling system using PCM and nanofluid for thermal regulation of concentrated photovoltaic solar cells. Energy Convers. Manag..

[12] Gholamibozanjani G., Farid M. (2020). A comparison between passive and active PCM systems applied to buildings. Renew. Energy.

[13] Kousksou T., Bruel P., Cherreau G., Leoussoff V., El Rhafiki T. (2011). PCM storage for solar DHW: From an unfulfilled promise to a real benefit. Sol. Energy.

[14] Haillot D., Franquet E., Gibout S., Bédécarrats J.-P. (2013). Optimization of solar DHW system including PCM media. Appl. Energy.

[15] Salih S.M., Jalil J.M., Najim S.E. (2019). Experimental and numerical analysis of double-pass solar air heater utilizing multiple capsules PCM. Renew. Energy.

[16] Mao Q., Yang M. (2020). Study on heat transfer performance of a solar double-slope PCM glazed roof with different physical parameters. Energy Build..

[17] Navarro L., de Gracia A., Castell A., Cabeza L.F. (2016). Experimental study of an active slab with PCM coupled to a solar air collector for heating purposes. Energy Build..

[18] Kong X., Wang L., Li H., Yuan G., Yao C. (2020). Experimental study on a novel hybrid system of active composite PCM wall and solar thermal system for clean heating supply in winter. Sol. Energy.

[19] Dordelly J.C.F., El Mankibi M., Roccamena L., Remion G., Landa J.A. (2019). Experimental analysis of a PCM integrated solar chimney under laboratory conditions. Sol. Energy.

[20] Ke W., Ji J., Xu L., Yu B., Tian X., Wang J. (2021). Numerical study and experimental validation of a multi-functional dual-air-channel solar wall system with PCM. Energy.

[21] Esen M. (2000). Thermal performance of a solar-aided latent heat store used for space heating by heat pump. Sol. Energy.

[22] Saffari M., de Gracia A., Ushak S., Cabeza L.F. (2017). Passive cooling of buildings with phase change materials using whole-building energy simulation tools: A review. Renew. Sustain. Energy Rev..

[23] Tyagi V.V., Buddhi D. (2007). PCM thermal storage in buildings: A state of art. Renew. Sustain. Energy Rev..

[24] Voelker C., Kornadt O., Ostry M. (2008). Temperature reduction due to the application of phase change materials. Energy Build..

[25] Jeong S.-G., Wi S., Chang S.J., Lee J., Kim S. (2019). An experimental study on applying organic PCMs to gypsum-cement board for improving thermal performance of buildings in different climates. Energy Build..

[26] Shukla N., Fallahi A., Kosny J. (2012). Performance characterization of PCM impregnated gypsum board for building applications. Energy Procedia.

[27] Shen Y., Liu S., Zeng C., Zhang Y., Li Y., Han X., Yang L., Yang X. (2021). Experimental thermal study of a new PCM-concrete thermal storage block (PCM-CTSB). Constr. Build. Mater..

[28] Entrop A.G., Brouwers H.J.H., Reinders A. (2011). Experimental research on the use of micro-encapsulated Phase Change Materials to store solar energy in concrete floors and to save energy in Dutch houses. Sol. Energy.

[29] Mahdaoui M., Hamdaoui S., Msaad A.A., Kousksou T., El Rhafiki T., Jamil A., Ahachad M. (2021). Building bricks with phase change material (PCM): Thermal performances. Constr. Build. Mater..

[30] Muthuvelan T., Panchabikesan K., Munisamy R., Nibhanupudi K.M., Ramalingam V. (2018). Experimental investigation of free cooling using phase change material-filled air heat exchanger for energy efficiency in buildings. Adv. Build. Energy Res..

[31] Li S., Zhu N., Hu P., Lei F., Deng R. (2019). Numerical study on thermal performance of PCM Trombe Wall. Energy Procedia.

[32] Kośny J. (2015). PCM-Enhanced Building Components; An Application of Phase Change Materials in Building Envelopes and Internal Structures.

[33] Duan S., Wang L., Zhao Z., Zhang C. (2021). Experimental study on thermal performance of an integrated PCM Trombe wall. Renew. Energy.

[34] Silva T., Vicente R., Rodrigues F., Samagaio A., Cardoso C. (2015). Development of a window shutter with phase change materials: Full scale outdoor experimental approach. Energy Build..

[35] Chan A. (2011). Energy and environmental performance of building façades integrated with phase change material in subtropical Hong Kong. Energy Build..

[36] Choi Y., Mae M., Kim H.B. (2019). Thermal performance improvement method for air-based solar heating systems. Sol. Energy.

[37] Gutherz J.M., Schiler M.E. (1991). A Passive Solar Heating System for the Perimeter Zone of Office Buildings. Energy Sources.

[38] Mousavi S., Rismanchi B., Brey S., Aye L. (2021). PCM embedded radiant chilled ceiling: A state-of-the-art review. Renew. Sustain. Energy Rev..

[39] Bouzennada T., Mechighel F., Filali A., Ghachem K., Kolsi L. (2021). Numerical investigation of heat transfer and melting process in a PCM capsule: Effects of inner tube position and Stefan number. Case Stud. Therm. Eng..

[40] Bouzennada T., Mechighel F., Ismail T., Kolsi L., Ghachem K. (2020). Heat transfer and fluid flow in a PCM-filled enclosure: Effect of inclination angle and mid-separation fin. Int. Commun. Heat Mass Transf..

[41] Bodin N.B., Semenov A.S., Shchegolkov A.V., Shchegolkovand A.V., Popova A.A. (2016). Nanomodified heat accumulating mate-rials for energy saving in industrial processes. Eco. Env. Cons..

[42] Shchegolkov A., Dyachkova T., Semenov A. (2017). The heat storage material based on paraffin-modified multilayer carbon nanotubes with Nickel-zinc ferrite. IOP Conf. Series Mater. Sci. Eng..

[43] Yu S., Jeong S.-G., Chung O., Kim S. (2014). Bio-based PCM/carbon nanomaterials composites with enhanced thermal conductivity. Sol. Energy Mater. Sol. Cells.

[44] Kenisarin M., Mahkamov K., Kahwash F., Makhkamova I. (2019). Enhancing thermal conductivity of paraffin wax 53–57 °C using expanded graphite. Sol. Energy Mater. Sol. Cells.

[45] Choi D.H., Lee J., Hong H., Kang Y.T. (2014). Thermal conductivity and heat transfer performance enhancement of phase change materials (PCM) containing carbon additives for heat storage application. Int. J. Refrig..

[46] Mazhar A.R., Shukla A., Liu S. (2020). Numerical analysis of rectangular fins in a PCM for low-grade heat harnessing. Int. J. Therm. Sci..

[47] Luo J., Zou D., Wang Y., Wang S., Huang L. (2022). Battery thermal management systems (BTMs) based on phase change material (PCM): A comprehensive review. Chem. Eng. J..

[48] Javadi F., Metselaar H., Ganesan P. (2020). Performance improvement of solar thermal systems integrated with phase change materials (PCM), a review. Sol. Energy.

[49] Arıcı M., Bilgin F., Nižetić S., Karabay H. (2020). PCM integrated to external building walls: An optimization study on maximum activation of latent heat. Appl. Therm. Eng..

[50] Mettawee E.-B.S., Assassa G.M. (2007). Thermal conductivity enhancement in a latent heat storage system. Sol. Energy.

[51] Wang P., Yao H., Lan Z., Peng Z., Huang Y., Ding Y. (2016). Numerical investigation of PCM melting process in sleeve tube with internal fins. Energy Convers. Manag..

[52] Baniassadi A., Sajadi B., Amidpour M., Noori N. (2016). Economic optimization of PCM and insulation layer thickness in residential buildings. Sustain. Energy Technol. Assess..

[53] Chopra K., Tyagi V., Pandey A., Sharma R.K., Sari A. (2020). PCM integrated glass in glass tube solar collector for low and medium temperature applications: Thermodynamic & techno-economic approach. Energy.

[54] Velmurugan K., Kumarasamy S., Wongwuttanasatian T., Seithtanabutara V. (2021). Review of PCM types and suggestions for an applicable cascaded PCM for passive PV module cooling under tropical climate conditions. J. Clean. Prod..

[55] Shamsi H., Boroushaki M., Geraei H. (2017). Performance evaluation and optimization of encapsulated cascade PCM thermal storage. J. Energy Storage.

[56] Jia X., Zhai X., Cheng X. (2019). Thermal performance analysis and optimization of a spherical PCM capsule with pin-fins for cold storage. Appl. Therm. Eng..

[57] Allouhi A., Msaad A.A., Amine M.B., Saidur R., Mahdaoui M., Kousksou T., Pandey A., Jamil A., Moujibi N., Benbassou A. (2018). Optimization of melting and solidification processes of PCM: Application to integrated collector storage solar water heaters (ICSSWH). Sol. Energy.

[58] Barzin R., Chen J.J., Young B.R., Farid M.M. (2016). Application of weather forecast in conjunction with price-based method for PCM solar passive buildings—An experimental study. Appl. Energy.

[59] Lu S., Gao J., Tong H., Yin S., Tang X., Jiang X. (2020). Model establishment and operation optimization of the casing PCM radiant floor heating system. Energy.

[60] Mehrpooya M., Hemmatabady H., Ahmadi M.H. (2015). Optimization of performance of Combined Solar Collector-Geothermal Heat Pump Systems to supply thermal load needed for heating greenhouses. Energy Convers. Manag..

[61] Mazhar A.R., Liu S., Shukla A. (2021). Numerical investigation of the heat transfer enhancement using corrugated pipes in a PCM for grey water harnessing. Therm. Sci. Eng. Prog..

[62] Afshan M.E., Selvakumar A., Velraj R., Rajaraman R. (2020). Effect of aspect ratio and dispersed PCM balls on the charging performance of a latent heat thermal storage unit for solar thermal applications. Renew. Energy.

[63] Saafi K., Daouas N. (2019). Energy and cost efficiency of phase change materials integrated in building envelopes under Tunisia Mediterranean climate. Energy.

[64] Zhu N., Wu M., Hu P., Xu L., Lei F., Li S. (2018). Performance study on different location of double layers SSPCM wallboard in office building. Energy Build..

[65] Jin X., Medina M.A., Zhang X. (2016). Numerical analysis for the optimal location of a thin PCM layer in frame walls. Appl. Therm. Eng..

[66] Heim D., Clarke J.A. (2004). Numerical modelling and thermal simulation of PCM & ndash; gypsum composites with ESP-r. Energy Build..

[67] Sovetova M., Memon S.A., Kim J. (2019). Thermal performance and energy efficiency of building integrated with PCMs in hot desert climate region. Sol. Energy.

[68] Qu Y., Zhou D., Xue F., Cui L. (2021). Multi-factor analysis on thermal comfort and energy saving potential for PCM-integrated buildings in summer. Energy Build..

[69] Alam M., Jamil H., Sanjayan J., Wilson J. (2014). Energy saving potential of phase change materials in major Australian cities. Energy Build..

[70] Wang H., Lu W., Wu Z., Zhang G. (2020). Parametric analysis of applying PCM wallboards for energy saving in high-rise lightweight buildings in Shanghai. Renew. Energy.

[71] Zhao J., Ji Y., Yuan Y., Zhang Z., Lu J. (2018). Energy-Saving Analysis of Solar Heating System with PCM Storage Tank. Energies.

[72] Devaux P., Farid M.M. (2017). Benefits of PCM underfloor heating with PCM wallboards for space heating in winter. Appl. Energy.

[73] Calise F., Cappiello F.L., D’accadia M.D., Vicidomini M. (2020). Dynamic modelling and thermoeconomic analysis of micro wind turbines and building integrated photovoltaic panels. Renew. Energy.

[74] Panayiotou G., Kalogirou S., Tassou S. (2016). Evaluation of the application of Phase Change Materials (PCM) on the envelope of a typical dwelling in the Mediterranean region. Renew. Energy.

[75] Mi X., Liu R., Cui H., Memon S.A., Xing F., Lo Y. (2016). Energy and economic analysis of building integrated with PCM in different cities of China. Appl. Energy.

[76] Solgi E., Memarian S., Moud G.N. (2018). Financial viability of PCMs in countries with low energy cost: A case study of different climates in Iran. Energy Build..

[77] Kosny J., Shukla N., Fallahi A. Cost Analysis of Simple Phase Change Material-Enhanced Building Envelopes in in Southern U.S. Climates. Report. 1 January 2013; Golden, Colorado. https://digital.library.unt.edu/ark:/67531/metadc838307/.

[78] Souayfane F., Biwole P.H., Fardoun F., Achard P. (2019). Energy performance and economic analysis of a TIM-PCM wall under different climates. Energy.

[79] Yun B.Y., Park J.H., Yang S., Wi S., Kim S. (2020). Integrated analysis of the energy and economic efficiency of PCM as an indoor decoration element: Application to an apartment building. Sol. Energy.

[80] Figueiredo A., Vicente R., Lapa J., Cardoso C., Rodrigues F., Kämpf J. (2017). Indoor thermal comfort assessment using different constructive solutions incorporating PCM. Appl. Energy.

